# Molecular characterization of Tunisian families with abetalipoproteinemia and identification of a novel mutation in *MTTP* gene

**DOI:** 10.1186/1746-1596-8-54

**Published:** 2013-04-04

**Authors:** Mohamed Najah, Sarraj Mohamed Youssef, Hrira Mohamed Yahia, Slimani Afef, Jelassi Awatef, Hammami Saber, Najjar Mohamed Fadhel, Agnès Sassolas, Slimane Mohamed Naceur

**Affiliations:** 1 Research Unit: UR 12ES09 Dyslipidemia and Atherogenesis, Faculty of Medicine, Monastir, Tunisia; 2 Research Unit 07/UR/06, Faculty of Pharmacy, Monastir, Tunisia; 3 Faculty of Medicine, Monastir, Pediatric Service, University Hospital of Monastir, Monastir, Tunisia; 4 Laboratory of Biochemistry and Toxicology of the University Hospital of Monastir, Monastir, Tunisia; 5 NSERM U1060 CarMeN, University of Lyon, Lyon, France

**Keywords:** Abetalipoproteinemia, Tunisian children, Mutations, *MTTP* gene

## Abstract

**Background:**

Abetalipoproteinemia (ABL; OMIM 200100) is a rare monogenic disorder of lipid metabolism characterized by reduced plasma levels of total cholesterol (TC), low density lipoprotein-cholesterol (LDL-C) and almost complete absence of apolipoprotein B (apoB). ABL results from genetic deficiency in microsomal triglyceride transfer protein (MTP; OMIM 157147). In the present study we investigated two unrelated Tunisian patients, born from consanguineous marriages, with severe deficiency of plasma low-density lipoprotein (LDL) and apo B.

**Methods:**

Intestinal biopsies were performed and The *MTTP* gene was amplified by Polymerase chain reaction then directly sequenced in patients presenting chronic diarrhea and retarded growth.

**Results:**

First proband was homozygous for a novel nucleotide deletion (c. 2611delC) involving the exon 18 of *MTTP* gene predicted to cause a non functional protein of 898 amino acids (p.H871I fsX29). Second proband was homozygous for a nonsense mutation in exon 8 (c.923 G > A) predicted to cause a truncated protein of 307 amino acids (p.W308X), previously reported in ABL patients.

**Conclusions:**

We discovered a novel mutation in *MTTP* gene and we confirmed the diagnosis of abetalipoproteinemia in new Tunisian families.

**Virtual slides:**

The virtual slide(s) for this article can be found here: http://www.diagnosticpathology.diagnomx.eu/vs/8134027928652779.

## Background

ABL is a rare autosomal recessive metabolic disorder characterized by very low levels of total cholesterol (TC), low density lipoprotein- cholesterol (LDL-C) and almost the complete absence of apolipoprotein B-containing lipoproteins [1]. Patients with ABL have severe fat malabsorption, retarded growth, lipid accumulation in hepatocytes and enterocytes, and peripheral blood acanthocytosis essentially in early childhood, and later develop a retinitis pigmentosa and neuropathy due to fat soluble vitamins deficiency specifically vitamin E. Other manifestations of ABL include also hepatic steatosis and hepatomegaly [2]. Heterozygotes ABL patients usually have normal plasma levels of TC and LDL-C [3].

ABL is due to mutations in the *MTTP* gene encoding for the microsomal triglyceride transfer protein (MTP) a protein of 97-kDa, containing 894 amino acids, that forms an heterodimer with the protein disulfide isomerase (PDI). MTP protein is essentially expressed in endoplasmic reticulum of hepatocytes and enterocytes and plays a key role on the assembly and secretion of apo B-containing lipoproteins in both liver and intestine [4-7].

The MTP molecule consists of three structural domains: N-terminal β-barrel (residues 22–297), α-helical (residues 298–603) and C-terminal domains (residues 604–894). The N-terminal β-barrel domain mediates the interaction with the N terminus of apo B; the middle α-helical domain mediates the interaction with both PDI and apo B; and the C-terminal mediates the lipid-binding and transfer catalytic activity of MTP [6,8,9].

To our knowledge, more than 50 patients with ABL were described in the literature. Most of *MTTP* gene mutations result in truncated proteins [10,11]. Missense mutations have been also reported in atypical cases of ABL and were associated with a milder form of the disease [12-14].

The aim of the present study was to characterize two unrelated Tunisian patients with the clinical manifestations and plasma lipid profile consistant with ABL. They were found to be homozygous for two mutations respectively, one of which is new in *MTTP* gene.

## Methods

### Clinical data and recruitment of family members

All subjects investigated are living in Tunisia and are of Tunisian ancestry. Informed consent was obtained from all adult subjects, and in the case of children, from their parents. The study was approved by the Ethics Committees of each participating institution. Family members (parents and their child) of the patients were asked to take part in the study.

### Patient II.1 (Family E)

**Figure 1:**
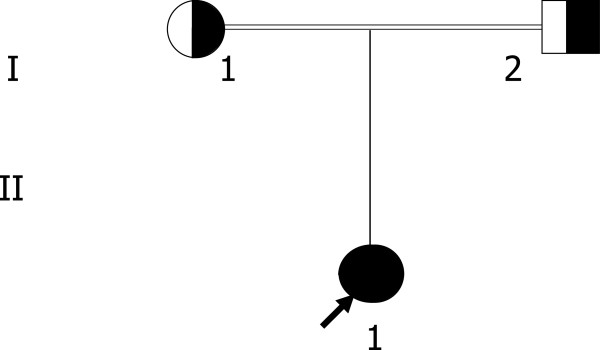
**Pedigree of Family E.** The proband is indicated by the arrow. Subject homozygote for the MTP gene mutation (c.2611delC) is indicated by close symbol, heterozygous carriers are indicated by half-shaded symbols.

The proband (subject II.1 in Figure 1) is a one year-old girl, born from a consanguineous marriage. She was referred to the hospital at the age of 10 months for the presence of hypotrophy and retarded growth. First observation showed that patient presented a steatorrhea (11.54 g/24 hours) with episodes of diarrhea. On physical examination, her weight was 3700 g and she had a height standard deviation score of −4.8 SDS.

Exploration showed that levels of ASAT and ALAT were 169 IU/L (normal values <38 IU/L) and 214 IU/L (normal values <41 IU/L) respectively. A normal level of gammaglutaryl transferase: 16 IU/L and an elevation of alcaline phosphatase (179 IU/L) (normal values 40–150 IU/L) were observed. Calcemia and total protein were normal. Acanthocytosis was revealed on peripheral blood smear. The lipid levels at the time of hospitalization were CT: 1.3 mmol/l, TG: 0.35 mmol/l and HDL-C: 0.9 mmol/l. Values of vitamins were 430 μg/l for vitamine A (normal values 444–945 μg/l), vitamine E (<1 μmol/l) was undetectable (normal values 16–35 μmol/l). The patient was treated by restricting dietary fat and by supplementation of fat soluble vitamin A 25 000 IU daily and vitamin E 500 mg daily during three months. No neurological or ophthalmic abnormalities were found.

### Patient II.2 (Family D)

**Figure 2:**
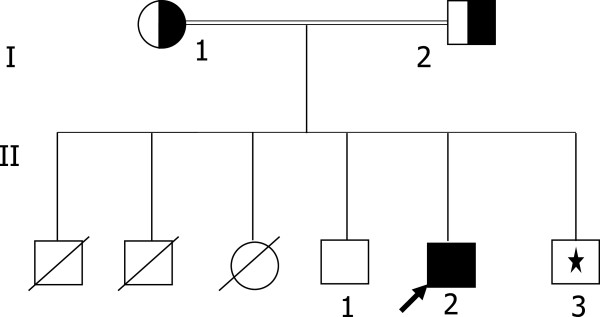
**Pedigree of Family D.** The proband is indicated by the arrow. Subject homozygote for the MTP gene mutation (c.923 G > A) is indicated by close symbol; heterozygous carriers are indicated by half-shaded symbols. Family member not available for the study is indicated by an asterisk.

The proband (subject II.2 in Figure 2) is a 20 year–old boy born from a consanguineous marriage. He suffered from a long history of sever hypobetalipoproteinemia. Clinical data showed that he was first referred to the hospital at the age of 5 months for the exploration of a prolonged diarrhea and retarded growth. Followed by oral and venous rehydration, antibiotherapy and a diet excluding proteins of cow’s milk; the evolution of the child was fluctuating. He left the hospital without any retained diagnosis; a low -fat diet was prescribed. The patient was present for a second examination at 4 years of age with chronic diarrhea and postprandial vomiting and retarded growth (−3DS). At this time a difficulty of the vision (twilight and night) and a disorder of walking were first observed. Anemia (Hb 8.9 mg/dl) and vitamine E deficiency (Vit E < 1 μmol/l) (normal values 16–35 μmol/l) were noted. Acanthocytosis was revealed on peripheral blood smear. At this age the diagnosis of familial hypobetalipoproteinemia was taken upon the low plasmatic lipids levels (CT: 1.28 mmol/l; TG: 0.31 mmol/l; apoB and LDL-C: undetectable). Ophthalmic examinations showed a peripheral hyperpigmentation of the retina. Neurological examinations revealed the presence of cerebellar syndrome and reduction in the osteotendinous reflexes. The patient was treated, during one year, by oral vitamin A 25 000 IU daily, vitamin E 800 mg daily, and vitamin K 40 mg every 15 days, the postprandial vomiting, walk and osteo-tendinous reflexes were normalized with a growth amelioration (−1DS). The other observations were not changed except the normalization of hemoglobin. Then, 5 mg daily of vitamin K were changed in his treatment and he was followed yearly until the age of 12 years. His health remained stable (Lipid values during follow-up were not documented in the clinical database). At age 20, he was hospitalized again, Neurological and ophthalmic examinations revealed a complete absence of osteo-tendinous reflexes, sensitive axonal neuropathy and pigmentary retinal degeneration. The hepatic ultrasound examination showed the presence of fatty liver. Plasma triglycerides and cholesterol were decreased, with undetectable LDL-C, ApoB and Vitamin E (Vit E <1 μmol/l). As the treatment was stopped for a long period the initial doses of vitamins were prescribed associated with a restricting dietary fat.

### Lipid and lipoprotein analysis

For all family members plasma lipid values were obtained after an overnight fast. Plasma TC, TG and HDL-C concentrations were measured by a standard method using the Synchrom CX7 clinical system (Beckman, Fullerton, CA, USA). LDL-C concentration was calculated using the Friedewald’s Formula. ApoA-I, apoB were measured by immunonephelometry using the IMMAGE immunochemistry system (Beckman, Fullerton, CA, USA).

### Intestinal biopsy

Following a period of 12 hours fasting, duodenal biopsies were obtained from probands. Tissues were fixed in 10% buffered formalin and embedded in paraffin for routine histology stained with hematoxylin and eosin.

### Molecular analysis

Genomic DNA was isolated from whole blood EDTA samples using the salting-out method [15]. For the family E patient, *MTTP* gene was amplified and directly sequenced as previously described [16]. In the case of family D, The promoter and all exons as well as flanking intronic sequences of the *MTTP* gene were amplified by polymerase chain reaction (PCR) as previously described [10], then PCR products were sequenced using BigDyeDeoxy terminator cycle sequencing reagents according to the manufacturer’s instructions and templates were sequenced on an automated ABI-PRISM 310 Genetic Analyzer (Applied Biosystems). Further, the *MTTP* mutations found in the probands were screened by direct sequencing in all family members available for study.

### Mutation nomenclature

*MTTP* mutations were designated according to the Human Genome Variation Society. Nucleotide numbers are derived from *MTTP* cDNA sequence (GenBank accession no. NM_000253.2), considering the A of first ATG translation initiation codon as nucleotide +1. Amino acid sequence MTP protein are described according to the National Center for Biotechnology Information reference sequence (MTP, GenBank accession no. NP_000244.2).

## Results

**Figure 3:**
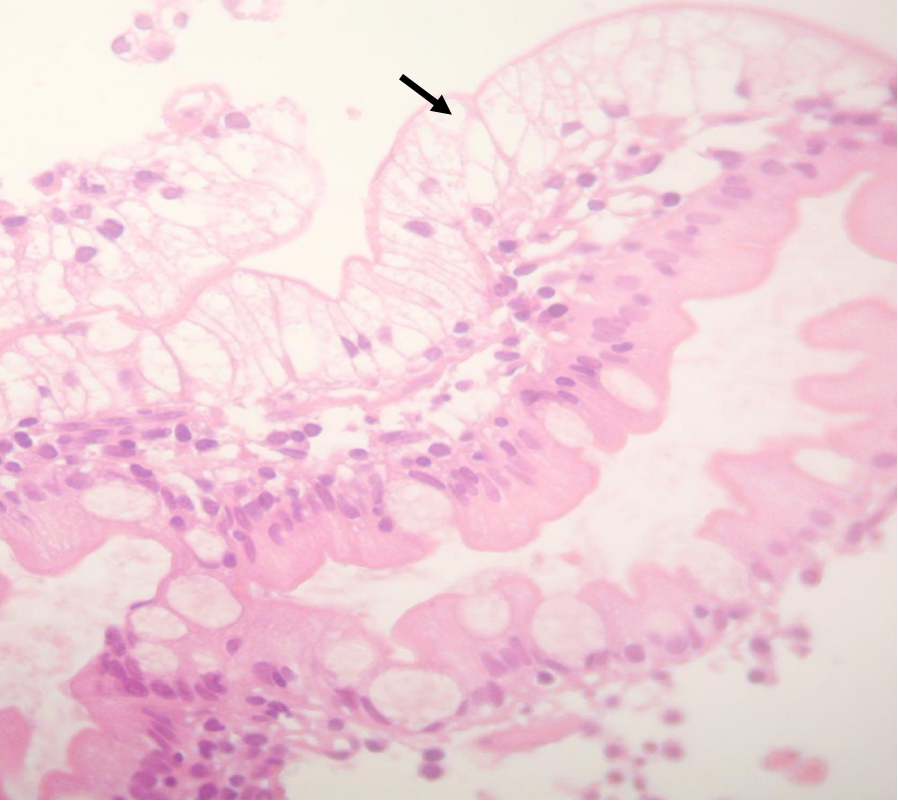
Light microscopy of the duodenal biopsy from the ABL patient of family E showed clusters of vacuolated enterocytes specifically at the tip of the villi (indicated by the arrow) (× 200 magnification).

The first proband of family E (subject II.1 in Figure 1 and Table 1) had extremely low plasma lipids with undetectable LDL-C and apoB (Table 1). As no data are available for the Tunisian population [10-34], the lipid values were compared to the 95th percentile of the levels found in the European populations suggesting a condition of hypobetalipoproteinemia [3]. The duodenal biopsy showed the presence of enterocyte vacuolization specifically at the tip of the villi suggestive of fat accumulation (Figure 3). Proband’s parents (Table 1) had a normal plasma lipid profile, strongly suggesting that the child might have ABL. The analysis of *MTTP* gene revealed that the proband was homozygous for a novel deletion in the exon 18 (c. 2611delC) expected to cause a frameshift in the mRNA by changing the sequence of the 23 last amino acids on C-terminal domain (residues 604–894) and adding 3 amino acids. The translation product of this abnormal mRNA is an MTP protein containing 898 amino acids (p.H871I fsX29) instead of 894 amino acids of the normal protein (Additional file 1). Proband’s parents were heterozygous.

**Table 1:** Plasma lipid and apolipoprotein levels in Family E

**Subject**	**Age (years)**		**TC**	**TG**	**LDL-C**	**HDL-C**	**ApoB**	**ApoA-I**
			Mmol/l				mg/dl	
I.1	33		4.39	1.47	2.6	1.16	70	178
I.2	38		5.46	1.09	3.08	1.63	87	191
II.1	1		0.58	0.14	und	0.37	und	49
		Normal range	3.9-6.7	0.68-1.7	2.5-4.4	>1.2	30-95	90-200

*TC*, total cholesterol; *TG*, triglyceride; *LDL-C*, low density lipoprotein-cholesterol; *HDL-C*, high density lipoprotein-cholesterol; *Apo*, apolipoprotein; *und*, undetectable.

**Figure 4:**
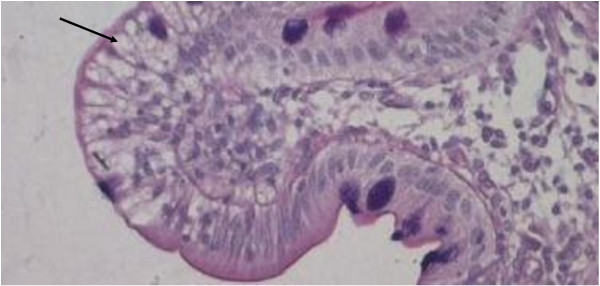
Light microscopy of the duodenal biopsy from the ABL patient of family D showed clusters of vacuolated enterocytes specifically at the tip of the villi (indicated by the arrow) (× 200 magnification).

The second proband of family D (subject II.2, in Figure 2 and Table 2) had extremely low plasma lipids. Plasma LDL-C and apoB were undetectable. The duodenal biopsy showed the presence of enterocyte vacuolization suggesting the presence of lipid droplets (Figure 4). Proband’s father (subject I.2 in Table 2) had a normal plasma lipid profile. Although the proband’s mother (subject I.1 in Table 2) had plasma levels of TC, LDL-C and apoB below the 95th percentile of the levels found in the European populations it was not possible to define to what extent the values observed in proband’s mother diverge from the mean in our population. The proband’s brother (subject II.1 in Table 2) had also low plasma lipids.

**Table 2:** Plasma lipid and apolipoprotein levels in Family D

**Subject**	**Age (years)**		**TC**	**TG**	**LDL-C**	**HDL-C**	**ApoB**	**ApoA-I**
			Mmol/l				mg/dl	
I.1	55		2.9	0.7	1.4	1.2	68	149
I.2	75		4.1	0.87	2.11	1.59	94	135
II.1	15		2.3	0.79	0.59	1.42	35	100
II.2	20		1.3	0.35	Und	0.9	und	35
		Normal range	3.9-6.7	0.68-1.7	2.5-4.4	>1.2	30-95	90-200

*TC*, total cholesterol; *TG*, triglyceride; *LDL-C*, low density lipoprotein-cholesterol; *HDL-C*, high density lipoprotein-cholesterol; *Apo*, apolipoprotein; *und*, undetectable.

In view of the lipid profile of proband’s parents and because we already described a Tunisian ABL family whith hypocholesteroleima in one of his parents [10], we assumed that the proband might be affected by ABL. The sequence of *MTTP* gene showed that the proband was homozygous for (c.923 G > A) mutation in exon 8. The result is a truncated protein of 307 amino acids, as opposed to the 894 amino acids, devoid of function. Proband’s parents were heterozygous and his brother (subject II.1 in Figure 2) present on this study was normal.

## Discussion

ABL is a rare autosomal recessive metabolic disorder characterized by complete absence of plasma apo B-containing lipoproteins that results from mutations in *MTTP* gene. Patients with ABL may develop severe neurological manifestations if they are not treated [2,17,18].

Abetalipoproteinemia is characterized, in term of clinical manifestations, with homozygous Familial Hypobetalipoproteinmia (FHBL; OMIM 107730) and Chylomicron Retention disease (CMRD; OMIM 246700), two other monogenic disorders of lipid metabolism by manifestations such as steatorrhea, retarded growth and chronic diarrhea associated with hypocholesterolemia. Under these conditions the use of the lipid profile of proband’s parents is essential to lead and facilitate the molecular diagnosis of these metabolic disorders: familial hypobetalipoproteinemia is a rare autosomal codominant disorder of lipoprotein metabolism, it results that both parents are expected to have plasma levels of TC, LDL-C and apoB lower than those found in normal subjects [3,19]. In ABL and CMRD with an autosomal recessive transmission, obligate heterozygotes have almost normal plasma lipoprotein profiles, with some exception and usually normal level of TG in patients homozygous for CMRD [2,20].

In this paper, we characterize two new Tunisian families with a severe form of hypobetalipoproteinemia. The common clinical features shared by patients were diarrhea, failure to thrive, acanthocytosis and lipid accumulation in enterocytes. The diagnosis of ABL was confirmed in these families and patients were found to be homozygotes for two mutations; one of them, which involves the exon 18, is novel on *MTTP* gene.

In the case of families E and D, probands presented with a similar lipid profile and very low plasma levels of TC, TG and undetectable LDL-C and apoB. In family E, the lipid values of proband’s parents, were within the normal range (Table. 1). The *MTTP* gene sequencing showed that proband of family E was homozygous for a novel mutation on *MTTP* gene (c.2611delC), predicted to result in a protein of 898 amino acids. It is well known that C-terminal domain (residues 604–894) mediate the lipid binding and the transfer catalytic activity of MTP [8,27]. The abnormal protein is expected to be non functional, since the sequence of the C-terminal domain was changed. It was demonstrated that C-terminal domain is preferentially conserved amongst vertebrates suggesting that this region might be critical for the triacylglycerol transfer activity associated with MTP [28]. The truncated MTP protein resulting from the mutation (p.G865X), involving the exon 18, was suggested to interfere with the association between the 97 kD subunit of MTP and PDI [24,29]. Other MTP truncations devoid of the C-terminal domain [25,30] or missense mutations located in this domain [12,13] have been described in patients with ABL highlighting the functional role of the C-terminal domain.

In family D, we found the third Tunisian patient with (c.923 G > A) mutation in homozygous state. This mutation was found for the first time in two Tunisian affected brothers (unrelated to the patient in the present study) and it leads to a truncated MTP protein of 307 amino acids (p.W308X) devoid of function [10]. The proband’s parents were heterozygous and his brother was normal. Proband’s mother (subject I.1 in Table 2) had low plasma lipid levels suggestive of hypobetalipoproteinemia, even though, the lipid plasma values within the lower limit or low values of TC and apoB were observed in heterozygous subjects for the same mutation (c.923 G > A) in a previous study [10]. This is not surprising as heterozygous ABL (specifically one of the parents) were reported with low plasma TC, LDL-C and apoB in other single families [21-23], and the possibility that human MTP deficiency is inherited in a codominant manner had been also suggested in heterozygous of MTP knockout mice with reduced plasma apoB levels [35].

The proband’s brother (subject II.1 in Table 2), with normal genotype, had low levels of TC and apoB although he was healthy and had no history of malabsorption or failure to thrive. The biochimical phenotype of family D strongly confirms the existence of a Tunisian specificity associated with monogenic dyslipidemias, since we recently showed a low level of plasma lipids in heterozygous for CMRD [36] and normal or moderate levels of CT and LDL-C in heterozygous familial hypercholestetolemia (FH) [37]. The lipid profile of proband’s brother (subject II.1 in Table 2) can be attributed either to the low content of fats in the mediterranean diet which characterize Tunisian population [38] or to the coexistence of other cholesterol-lowering gene(s) associated with hypobetalipoproteinemia in this family. In addition, it has been shown that some polymorphisms in genes related to the metabolism of lipoproteins, have been associated with lipid levels in Tunisian patients affected by other diseases like cardiovascular pathologies [39]. This reflects, in part, the genetic background heterogeneity of the Tunisian population demonstrated in other genetic disorders [40].

Many mutations in *MTTP* gene described in literature as cause of ABL were private and related to single families. There was an exception with (p.G865X) mutation which was found in ABL patients from USA and Canada and was also reported in the Ashkenazi Jewish population with a high prevalence and a founder effect [12,25]. The (419insA) mutation was also associated with ABL in patients of European ancestry [13,26]. In Tunisian population, four mutations in *MTTP* gene (c.923 G > A, c.619-3 T > G, c.1236 + 2 T > G and c.2342 + 1 G > A) have been reported as cause of ABL in four single families [10,36]. In this study, the diagnosis of the mutation (c.923 G > A) in a new Tunisian family is suggestive of a high prevalence of the mutant allele in heterozygote state.

Although, the clinical data available were rather incomplete (especially hepatic exploration), our patients presented the typical hematologic and gastrointestinal features of ABL such as acanthocytosis, diarrhea during infancy and malabsorption with mild anemia (proband II.2 of family D in this paper) which was reported in some cases of ABL [10,21]. Usually neurological manifestations begin later in life in ABL patients, especially those who have not been given a supplementation of vitamin E [31]. Later supplementation of fat soluble vitamins in patient II.2 (family D) allowed the osteotendinous reflexes reduction. Previous studies showed that early therapy with vitamin E, before the age of 16 months prevents neurologic dysfunction [32,33]. Elevated Transaminases serum of the proband II.1 (family E) is suggestive of hepatic steatosis which is needed to be confirmed by liver biopsy in this patient. It was reported that hepatic manifestations in ABL cases included hepatomegaly due to hepatic steatosis in association with elevated transaminases [34,41]. The absence of neurological and opthalmological manifestations in patient II.1 (family E) is consistant with the observation that the diagnosis of ABL should be made in children with malabsorption, acanthocytosis and hypocholesterolemia. Indeed a low-fat diet and fat-soluble vitamins supplementation can prevent later complications [17].

## Conclusion

Five ABL patients have been reported in Tunisian population [10,36]. In this work we characterize two other unrelated patients affected by this metabolic disorder and we identify a novel mutation in *MTTP* gene leading to a non functional protein. Although it is a rare disease, abetalipoproteinemia seems to have a high prevalence in Tunisia. Knowledge of *MTTP* gene mutations allows us to set up a sensitive and specific use of genetic testing for screening carriers in other families.

## Abbreviations

ABL: Abetalipoproteinemia; ApoA-I: Apolipoprotein A-I; ApoB: Apolipoprotein B; CM: Chylomicrons; CMRD: Chylomicron Retention Disease; FHBL: Familial Hypobetalipoproteinemia; HDL-C: High density lipoprotein–cholesterol; LDL-C: Low density lipoprotein–cholesterol; mRNA: Messenger RNA; MTP: Microsomal triglyceride transfer protein; MTTP: Microsomal triglyceride transfer protein large subunit gene; PCR: Polymerase chain reaction; PDI: Protein disulfide isomerase; TC: Total cholesterol; 1TG: Triglyceride.

## Competing interests

The authors declare that they have no competing interests.

## Authors’ contributions

NM wrote the manuscript. SMY and HMY participated in data analysis. AS and SMN revised the manuscript and save final approval of the version to be published. All authors read and approved the final manuscript.

## Supplementary Material

Additional file 1 **Analysis of the *MTTP *gene.** The chromatogram show the partial sequence of exon 18 in the proband II.1 (family E). In above, the normal *MTTP* gene sequence (reference NM_000253). In the below, mutant sequence show the homozygous state of the novel nucleotide deletion (c. 2611delC) and the frameshift in the mRNA. The predicted translation product of the mutant *MTTP* gene is a non functional protein of 898 amino acids (p.H871I fsX29) lacking the last 23 functional amino-acids. Click here for file
