# Supplementary material for: Molecular characterization of Tunisian families with abetalipoproteinemia and identification of a novel mutation in MTTP gene
Source: Diagn Pathol. 2013 Apr 4;8:54. doi: 10.1186/1746-1596-8-54 (PMC3632489; doi:10.1186/1746-1596-8-54)
Supplement: Additional file 1 — Analysis of the MTTP gene. The chromatogram show the partial sequence of exon 18 in the proband II.1 (family E). In above, the normal MTTP gene sequence (reference NM_000253). In the below, mutant sequence show the homozygous state of the novel nucleotide deletion (c. 2611delC) and the frameshift in the mRNA. The predicted translation product of the mutant MTTP gene is a non functional protein of 898 amino acids (p.H871I fsX29) lacking the last 23 functional amino-acids. [file 1746-1596-8-54-S1.ppt]

## Slide 1
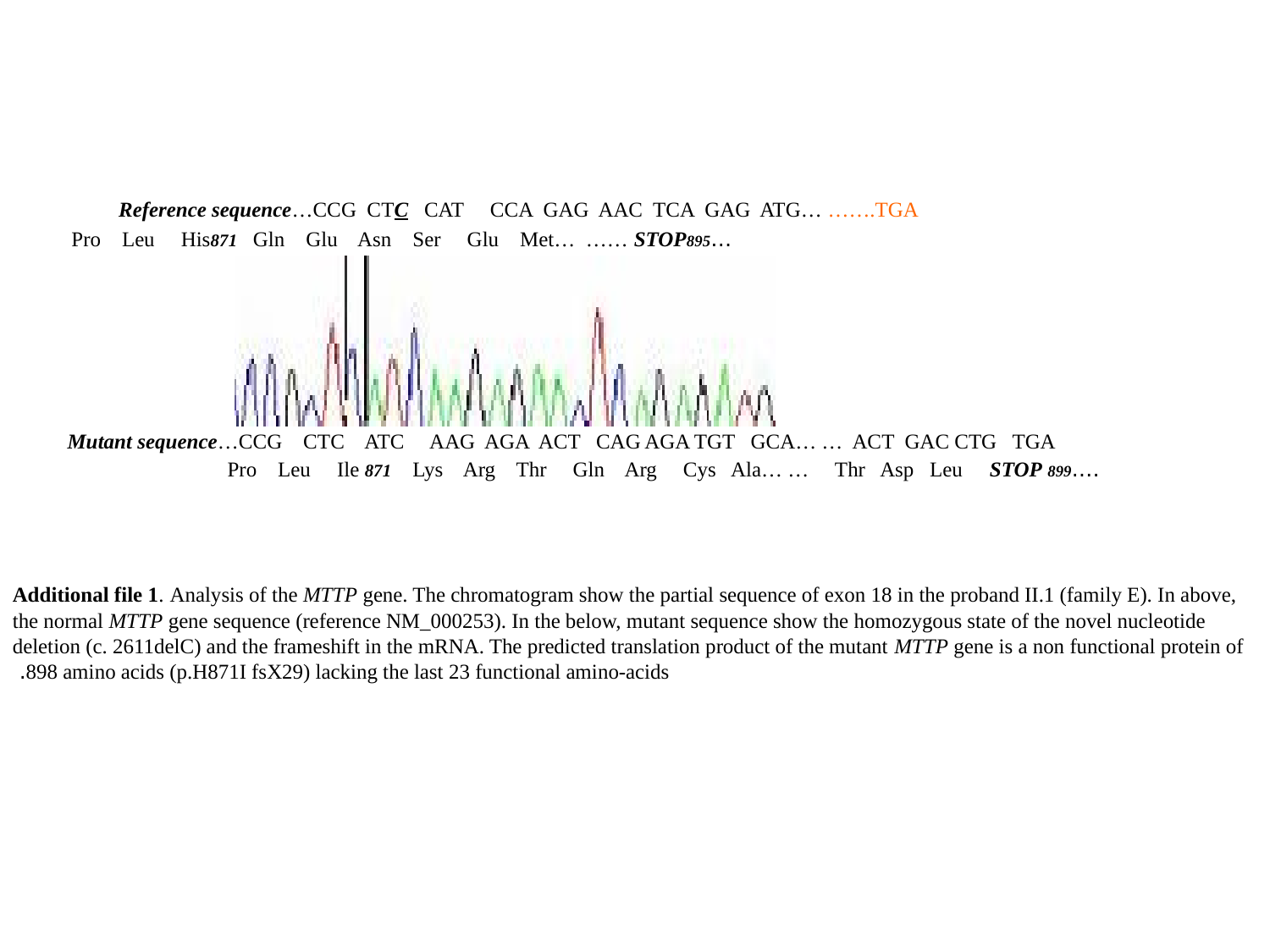

Reference sequence…CCG CTC CAT CCA GAG AAC TCA GAG ATG… …….TGA
 …Pro Leu His871 Gln Glu Asn Ser Glu Met… …… STOP895
# Mutant sequence…CCG CTC ATC AAG AGA ACT CAG AGA TGT GCA… … ACT GAC CTG TGA
	….Pro Leu Ile 871 Lys Arg Thr Gln Arg Cys Ala… … Thr Asp Leu STOP 899
Additional file 1. Analysis of the MTTP gene. The chromatogram show the partial sequence of exon 18 in the proband II.1 (family E). In above, the normal MTTP gene sequence (reference NM_000253). In the below, mutant sequence show the homozygous state of the novel nucleotide deletion (c. 2611delC) and the frameshift in the mRNA. The predicted translation product of the mutant MTTP gene is a non functional protein of 898 amino acids (p.H871I fsX29) lacking the last 23 functional amino-acids.
